# Cardiorespiratory Fitness, Blood Pressure and Ethnicity Are Related to Salivary Cortisol Responses after an Exercise Test in Children: The ExAMIN Youth SA Study

**DOI:** 10.3390/ijerph18157898

**Published:** 2021-07-26

**Authors:** Sabrina Köchli, Shani Botha-Le Roux, Aletta Sophia Uys, Ruan Kruger

**Affiliations:** 1 Hypertension in Africa Research Team (HART), Faculty of Health Sciences, North-West University, Potchefstroom 2520, South Africa; sabrina.koechli@gmx.ch (S.K.); shani.botha@nwu.ac.za (S.B.-L.R.); lisa.uys@nwu.ac.za (A.S.U.); 2 Medical Research Council, Unit for Hypertension and Cardiovascular Disease, Faculty of Health Sciences, North-West University, Potchefstroom 2520, South Africa

**Keywords:** salivary cortisol, childhood obesity, physical fitness, ethnicity

## Abstract

Background: Childhood elevated circulatory stress mediators such as cortisol seem to play an important role in the development of hypertension and metabolic disorders later in life. Little is known about the association of body composition, cardiorespiratory fitness (CRF), blood pressure (BP) and ethnicity with cortisol reactivity in young children. Methods: In this cross-sectional study, 324 black and 227 white school children (aged 7.4 ± 1.0 years) were screened for salivary cortisol reactivity, body mass index, BP and CRF (shuttle run) by standardised assessments for children. Results: Children in the lower cortisol reactivity percentile (<25th) had a higher heart rate (87.0 ± 12.9 bpm) and a lower CRF (3.1 ± 1.3 stages) compared to children in the upper (>25th) percentile (86.2 ± 11.5 bpm and 3.5 ± 1.7 stages, respectively). At baseline, children of black ethnicity had a higher cortisol level (*p* < 0.001). Immediately before the exercise test, no associations of obesity, BP, CRF and ethnicity with cortisol levels were found. In analysis of covariance (ANCOVA) we found that low CRF, high BP and black ethnicity were independently associated with lower cortisol reactivity by performing the shuttle run test (*p* < 0.01). Conclusion: Low CRF and high BP were associated with lower cortisol reactivity after a cardiorespiratory exercise test. Black children showed a lower cortisol reactivity which may contribute to the earlier onset of hypertension reported in black compared to white populations. Primary prevention programs need to focus on improving physical fitness to reduce the growing prevalence of cardiometabolic disorders during childhood.

## 1. Introduction

Chronic stress, defined as a perceived disruption of homeostasis over time, leads to a variety of physiological maladaptive alterations such as hypertension [1], obesity [2] and type 2 diabetes mellitus [3] in adults [4]. Based on the concept of allostasis [5], acute exposure to environmental, emotional and physical stressful stimuli contributes to a normal and healthy development of metabolic and cardiovascular function during childhood [6,7]. However, chronic stress-related elevation in circulatory mediators in children seem to play an important role in the development of hypertension and metabolic disorders later in life [8,9]. Cortisol reactivity is an index of the hypothalamic-pituitary-adrenal axis (HPA) and autonomic nervous system activity [3,4,5]. The HPA cascade stimulates adrenal glands to release cortisol, which leads to a number of metabolic effects, such as an increase in blood pressure (BP), cardiac output and circulating glucose levels [7]. Growing evidence suggests that persistent higher cortisol concentrations are responsible for hypersecretion of insulin and growth factors which may lead to a maladaptive accumulation of visceral adipose tissue, dyslipidaemia and elevated BP [10,11]. Data on the association of obesity and high BP with cortisol in children are scarce and inconsistent. Wirix et al. found that salivary cortisol is not associated with elevated BP in overweight and/or obese children [12]. A recent study demonstrated a lower morning cortisol level and a higher evening cortisol level in children with obesity compared to children with normal weight [13]. The same study found an inverse association between salivary cortisol level and time spent on moderate to vigorous physical activity. In adults, physical fitness and daily physical activity have the potential to reduce cortisol secretion during psychosocial distress conditions [14]. There are few studies on the relationship between cortisol and physical fitness in children. A previous study reported increased salivary cortisol levels after a cardiorespiratory fitness (CRF) assessment in prepubertal children, which suggests a correlation between environmental stressors and physiological responses after induced physical stress [15].

Although cardiovascular consequences of stress seem to be consistent across various ethnicities, [16] higher psychosocial distress has previously been demonstrated in black compared to white populations from the same region as the current study [17]. However, it remains unclear whether there are also ethnic-specific differences in cortisol reactivity and accumulation in response to an external stressor in children. We hypothesise that cardiovascular risk factors such as obesity, high blood pressure, low physical fitness and black ethnicity are associated with lower cortisol reactivity early in life. Our study, for the first time, aimed to examine the association of body composition and BP as well as physical fitness and ethnicity on salivary accumulation of cortisol at baseline, before and after a maximal cardiorespiratory endurance test in healthy prepubertal children.

## 2. Methods

### 2.1. Study Setting

The large scale, observational “The Exercise, Arterial Modulation and Nutrition in Youth South Africa” (ExAMIN Youth SA, Australia) study [18] was designed as a multidisciplinary, school-based cohort study in children. The detailed study design is described elsewhere [18]. Briefly, 5- to 9-year-old primary school children were screened based on current clinical childhood conditions such as obesity, blood pressure physical fitness and salivary cortisol analyses were performed. Screening took place in the morning on-site in a regular school setting and all children had to fast on the day of examination. Parents were requested to give permission for their child to participate, while children under 7 years gave written assent and children over 7 years gave written consent to participate. The study was approved by the Health Research Ethics Committee of the North-West University in Potchefstroom (NWU-00091-16-A1) and registered at ClinicalTrials.gov (NCT04056377). In this study, the Strengthening the Reporting of Observational Studies in Epidemiology (STROBE) guidelines were applied [19]. All assessments and procedures were performed according to the Guidelines for Good Clinical Practice [20].

### 2.2. Participants

**Figure 1:**
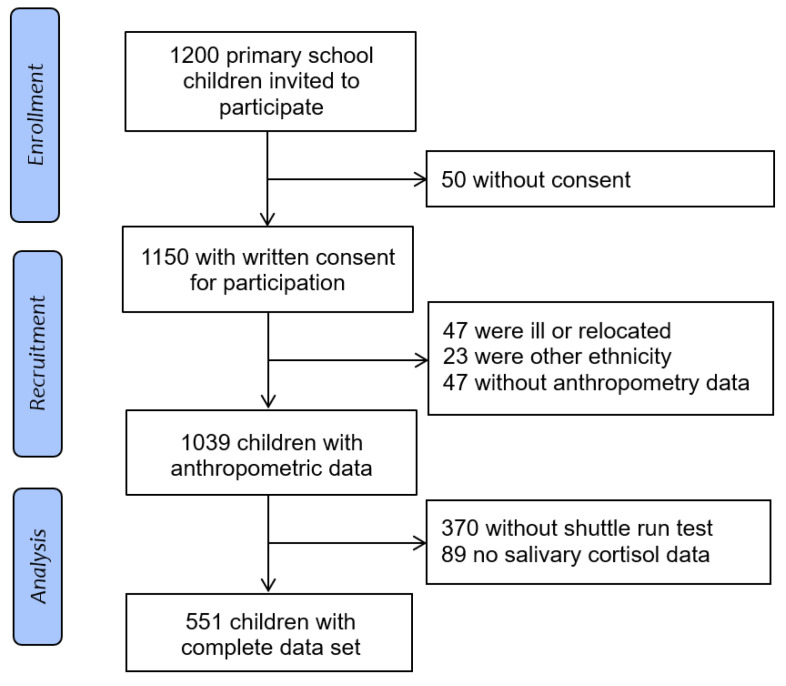
Flow diagram of the study population.

Overall, 1200 children were invited and 1150 (96%) provided permission and a written assent or consent to participate in the study. Forty-seven children dropped out because of illness or were absent from school, 23 children were coloured or of another ethnicity, 47 children did not have anthropometry data, 370 did not perform the shuttle run test and 89 children had no valid salivary cortisol data. Finally, 324 (59% black and 227 41% white) children had complete data (Figure 1).

### 2.3. Measurements

#### 2.3.1. Salivary Cortisol

Cortisol concentrations were assessed from saliva samples in SaliCaps^®^ (IBL International GmbH, Hamburg, Germany). The assessment of salivary cortisol levels allows for non-invasive and validated analyses of stress-induced cortisol accumulation in children and it is highly correlated with cortisol levels in blood serum [10,21]. Saliva samples were collected in a fasting state in the morning as a baseline measurement before 8 a.m., a second sample 30 min after the baseline sampling and a final sample 30 min after the CRF test (shuttle run performance). For further analysis, the salivary cortisol levels were determined using solid phase enzyme-linked immunosorbent assays (ELISA) with the Synergy HT microplate reader, BioTek^®^ Instruments, Inc. (Winooski, VT, USA). Changes in cortisol levels from pre- to post-shuttle run were calculated to assess cortisol reactivity to an external physical stressor.

#### 2.3.2. Sociodemographic Data and Anthropometry

A general health and sociodemographic questionnaire was used to collect personal data regarding socioeconomic status (SES), age, sex and ethnicity. Body height was measured with a wall-mounted stadiometer (Seca 213, Birmingham, UK) to the nearest 1 mm, weight with a digital scale to the nearest 0.1 kg and percentage body fat (BF) with the bioelectrical impedance analysis using the Bodystat 1500^®^ MDD (MultiScan 5000, BodyStat, Ltd. Douglas, Isle of Man). Waist circumference was taken in triplicate at the narrowest point between the lower costal border and the top of the iliac crest using the Lufkin Executive thinline 2 mm steel tape (Apex Tool Group B.V.; AK Emmen, The Netherlands) with an accuracy of 1 mm. All measurements were performed in light sport clothing without shoes according to the International Society for the Advancement of Kinanthropometry (ISAK) procedures [22]. Measurements of body composition were performed in the morning by 8 a.m. and all children were requested to fast on the day of examination. Children were categorised into clinically relevant body composition groups based on cut off points for sex-adjusted BMI z-scores for age according to reference values from the World Health Organisation (WHO). Children with a BMI above the 85th percentile in their sex and age group were categorised as being overweight and above the 95th percentile as children with obesity. Waist to height ratio (WHtR) was calculated to assess abdominal obesity [23]. A WHtR above 0.5 was reported to be predictive of future cardio-metabolic risk in children and adults [23,24].

#### 2.3.3. Blood Pressure

Systolic and diastolic BP were measured with appropriately sized cuffs for children and an automated oscillograph (Omron HBP-1100-E; OMRON HealthCare Co., LTD. Kyoto, Japan) was used to reduce inter-observer variability. Before the measurements, children had to sit quietly for five minutes with their feet on the floor. Five measurements were performed with 1-min resting intervals at a single visit. For statistical analysis and clinically relevant BP categorisation, the mean of the three measurements with the smallest variation was calculated. We categorised BP ranges according to the Clinical Practice Guidelines of the American Academy of Pediatrics, based on the WHO reference cohort [20]. Children with a BP between the 90th and 95th percentiles, were categorised as having elevated BP and above the 95th percentile, as BP in the hypertensive range.

#### 2.3.4. Cardiorespiratory Fitness

To assess maximal cardiorespiratory endurance, a 20 m shuttle run was performed. CRF was assessed in the morning during school hours with the same equipment and testing team for every school. After a 5 min warm-up, the well-established and validated CRF test was performed [25,26]. In this progressive endurance test, children had to run back and forth in a 20 m distance with an initial running speed at 8.0 km/h. After every minute, the running speed was increased by 0.5 km/h, paced by an audio system for the timing of the shuttle run test. The individual maximum stage was achieved when the child did not cross the 20 m line at the moment of the beep for two consecutive 20 m trials. All participants were verbally encouraged to perform at their maximum during each assessment. The score was counted as the number of stages (1 stage ≈ 1 min) reached with a precision of 0.5 stages. To assess physical fitness levels, children were categorised according to CRF tertiles to compare cortisol concentrations across the first and third tertiles.

### 2.4. Statistical Analysis

Variance homogeneity was assessed using Tukey-Anscombe plots. To assess normality, we inspected normal QQ plots for residuals. Logarithmic transformation was used to transform skewed cortisol data to normal distribution. The sample characteristics were presented in terms of cortisol reactivity percentiles. The lower 25th percentile (<p25) for reduced cortisol reactivity in the total cohort of children was used as the cut-off point. Early vascular ageing is stratified by the upper centiles of arterial stiffness, whereas reduced HPA-axis reactivity can be represented by the lower percentiles of cortisol levels. Lifestyle and behavioural risk factors including physical inactivity, abdominal obesity, high blood pressure, psychosocial stress as well as subsequent pro-inflammation and oxidative stress, impacts on HPA-axis reactivity and early vascular ageing [27]. Log-transformed cortisol concentration was analysed across the clinical categories of BMI, WHtR and BP, between ethnic groups and across tertiles of physical fitness using univariate analysis of covariance (ANCOVA) and p for trend values were reported. For analyses and graphics, an up-to-date version of Stata 15 (StataCorp LP, College Station, TX, USA) was used. The sample size was calculated with a strong effect size of f^2^ = 0.01 for a given 2-sided level alpha error of probability at 0.05 based on ten predictors for ANCOVA, a power of 95% was achieved.

## 3. Results

### 3.1. Population Characteristics and Prevalence

Population characteristics of the total cohort are shown in Table 1. Children with a lower cortisol reactivity (lower 25th percentile) had a higher heart rate (*p* = 0.009) and a lower shuttle run performance (*p* = 0.041), as well as a higher cortisol concentration at baseline/before the exercise test (*p* < 0.001) and a lower cortisol level after the shuttle run assessment (*p* < 0.001) compared to children above the 25th percentile.

In our cohort of black and white children, 85% (*n* = 467), were categorised as participants with normal weight, 8% (*n* = 44) with overweight and 7% (*n* = 40) as being children with obesity (Table 2). According to clinically relevant BP categories, 69% (*n* = 381) were in the normotensive range, 11% (*n* = 60) in the range of elevated BP and 20% (*n* = 110) in the hypertensive range. Black children showed a lower BMI (15.6 ± 2.1 kg/m^2^), but a higher percentage body fat (25.3 ± 7.4%) and a better shuttle run performance (3.4 ± 1.6 stages) compared to their white counterparts (BMI: 16.8 ± 3.0 kg/m^2^; shuttle run: 3.3 ± 1.6 stages; body fat: 23.5 ± 5.5%; *p* < 0.01).

### 3.2. Group Differences

**Figure 2:**
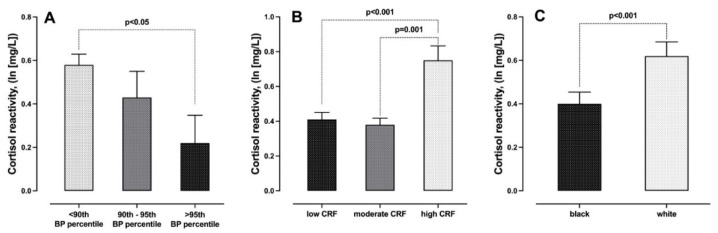
Comparison of cortisol reactivity in relation to (**A**) blood pressure categories, (**B**) tertiles of cardiorespiratory fitness and (**C**) black and white participants.

The results for between group differences are shown in Table 2. Children with a WHtR higher than 0.5 had a lower cortisol concentration at baseline compared to children with a WHtR of less than 0.5 (*p* < 0.05), but no significant trend was observed for BMI z-score categorisation. Children categorised as having elevated BP or within the hypertensive BP range showed a lower cortisol reactivity, i.e., lower changes in cortisol concentrations from pre- to post-shuttle run, compared to children with normal BP (*p* < 0.01) (Figure 2A). Low physical fitness was independently associated with lower post cortisol concentrations as well as lower cortisol reactivity across the CRF tertiles (*p* < 0.01) (Figure 2B). Black ethnicity was related to lower salivary cortisol at baseline and post shuttle run test and a lower cortisol reactivity compared to the white ethnic group (*p* < 0.01) (Figure 2C).

## 4. Discussion

Our hypothesis of obesity, high blood pressure, low cardiorespiratory fitness as well as black ethnicity relating unfavourably to lower cortisol reactivity in children was partially accepted. In line with our hypothesis, lower physical fitness and higher blood pressure associated with lower cortisol reactivity in young children. Baseline cortisol was inversely associated with abdominal obesity and a higher cortisol concentration was found in white children. Lower cortisol reactivity was observed in black compared to white children.

### 4.1. Physical Fitness and Cortisol

Cortisol is a well-known stress-related biomarker, also in paediatric studies, which is regulated by diurnal biorhythms and secreted by the HPA when triggered by environmental conditions and external stressors [28]. We demonstrated that high physical fitness is associated with a better cortisol reactivity in young children, independent of ethnicity and BMI. Children in the lower 25th percentile of cortisol reactivity showed a poorer shuttle run performance. No association between physical fitness, obesity or BP with salivary cortisol levels was found immediately before the shuttle run test. In line with our findings, a previous study showed an increase in salivary cortisol after a short running and/or cycling test in children [15]. The authors found that immediately after the exercise test, salivary cortisol accumulation remained unchanged and increased after 15 min of recovery. Another study found that sedentary behaviour, based on more than three hours screen time per day, was associated with a smaller increase in cortisol after wake-up time compared to children with less than three hours screen time per day [29]. Better physical fitness and less sedentary behaviour seem to play a key role in mediating favourable physical adaptations such less daily hassles and the ability to cope with to external stressors [30].

### 4.2. Obesity and Cortisol

A WHtR above 0.5 or excess abdominal adipose tissue was independently associated with lower morning cortisol levels at baseline measurement. Obesity was inversely associated with baseline cortisol, but not independent of ethnicity, BP or physical fitness. Similar to our findings, previous research demonstrated a lower morning cortisol accumulation in children with obesity and higher body fat [12,13]. However, others found a positive association of salivary and hair cortisol with obesity in young girls [31]. In our cohort of young children, no association between WHtR, obesity and cortisol reactivity after the shuttle run test was found. It is therefore possible that obesity will affect acute cortisol secretion only after a longer exposure time to increased adipose tissue.

### 4.3. Blood Pressure and Cortisol

Similar to our findings, a previous study found no association between cortisol parameters and hypertension in children with overweight or obesity at baseline measurement [12]. However, we further demonstrated that salivary cortisol reactivity was higher in children with a normal office BP compared to children with elevated BP or BP in the hypertensive range. It is well known that CRF is associated with favourable cardiovascular and mental health in children and adults [32,33,34]. Based on our results, higher BP and lower CRF contribute to a lower systemic cortisol reactivity during a cardiorespiratory exercise performance test. However, others found that higher cortisol reactivity after an externally induced negative mental stressor seems to be associated with the development of hypertension in adults [35]. In contrast, there is growing evidence indicating that reduced cardiovascular reactivity such as systolic BP and heart rate variability, is related to an increased risk to become obese and higher psychological stress [36,37]. In line with this assumption, we further found that heart rate at baseline was higher in children with lower cortisol reactivity (lower 25th percentile) compared to children with higher cortisol accumulation after the shuttle run test. Unfortunately, heart rate variability was not assessed in our study. Longitudinal studies in children may help to further differentiate the association of cortisol reactivity and cardiovascular outcomes later in life.

### 4.4. Ethnicity and Cortisol

Higher psychosocial stress seems to be more pronounced in black populations and therefore, a higher risk for cardiovascular alterations in black compared to white populations has been reported [17]. However, the association of ethnicity with cortisol reactivity in young children remains widely unexplored. In our cohort, black children had an unfavourable lower cortisol level, both at baseline and after the shuttle run performance, compared to white children. In addition, black children presented with a lower cortisol reactivity during the CRF test, which may contribute to a higher cardiovascular risk profile in black compared to white children. This is in line with previous studies which have shown that apparently healthy black children had increased arterial stiffness and carotid intima–media thickness compared to their white counterparts [38]. Physiological adaptations through higher cortisol reactivity may be a key mechanism to assess systemic cardiometabolic health early in life.

### 4.5. Potential Mechanisms

It is known that cortisol, the glucocorticoid hormone, has anti- and pro-inflammatory effects and is linked to increased blood levels under chronic inflammatory conditions [39,40]. The release of cortisol from the HPA axis in response to an acute external stressor may result in anti-inflammatory effects to counterbalance inflammation [40,41]. Lower cortisol reactivity may be responsible for declined immunological functions and increased levels of pro-inflammatory cytokines and the development of vascular impairments. Consistently increased stress mediators and the release of cortisol from the HPA axis lead to hypersecretion of insulin and the adipocyte hormone leptin, which may further trigger inflammatory and sympathovagal imbalance [11,42,43]. Exercise and physical fitness have the potential to reduce inflammatory stress parameters and attenuate cortisol secretion in response to psychosocial stress [44,45,46]. Acute activation of the stress system by physical exercise may be a key mechanism for normal cardiovascular development during childhood [6,7]. We hypothesise that higher cortisol reactivity during a positive external stressor (e.g., vigorous exercise) is associated with favourable cardiometabolic health in children. A balanced activation and regulation of the HPA axis and the sympathoadrenal system may be achieved by physical activity and exercise interventions to reduce high BP and the development of psychosocial distress early in life.

### 4.6. Strengths and Limitations

This was a cross-sectional analysis and we did not investigate the long-term associations of cortisol reactivity and cardiometabolic health during the life course. However, a significant and independent relationship between CRF, elevated BP, ethnicity and cortisol reactivity in children was found and therefore, a follow-up study is warranted to prove causal associations later in life. BP measurements took place in a regular school setting and therefore, the diagnosis of chronic high BP was not included in the study approach. Great care was taken for the assessment of office BP status in accordance with the recommendations of the American Heart Association [47]. In our cohort, only seven percent were children with obesity. Future investigations should include a higher number of children with obesity to further differentiate cortisol reactivity in black and white children related to body composition. One strength of our study includes the large sample size and the inclusion of children with black and white ethnicity. Another strength is the use of standardised procedures to measure body composition, BP, CRF and cortisol reactivity in young children.

## 5. Conclusions

In conclusion, our results demonstrate that lower physical fitness and higher BP are associated with lower cortisol responses in young children. Black children seem to have a lower acute cortisol reactivity and an unfavourable lower morning cortisol level compared to white counterparts. Children with a lower cortisol reactivity may have a higher probability of developing early vascular aging and subsequent arterial stiffness at much younger ages in adolescence or early adulthood. It remains to be shown whether circulating cortisol may be a sensitive biomarker of cardiometabolic risk during childhood until adulthood. Future intervention programs should focus on improving physical fitness in black and white children to achieve the ambitious long-term goal of reducing cardiovascular risk, especially in black populations.

## Figures and Tables

**Table 1:** Population characteristics of the study at baseline, according to under and upper cortisol reactivity percentiles.

Parameter	Total (*n* = 551)	Cortisol Groups		*p* Value
		<P25 (*n* = 137)	≥P25 (*n* = 414)	
Age	7.4 ± 1.0	7.2 ± 0.9	7.4 ± 1.0	0.118
Sex, boys (*n*, %)	242; 43.9	66; 12.0	176; 31.9	0.248
Ethnicity, black (*n*,%)	324; 58.8	94; 17.1	230; 41.7	0.007
Height (cm)	122.0 ± 8.2	121.1 ± 7.8	122.2 ± 8.3	0.152
Weight (kg)	24.3 ± 6.4	23.7 ± 5.8	24.4 ± 6.6	0.261
Body mass index (kg/m^2^)	16.1 ± 2.6	16.0 ± 2.5	16.1 ± 2.7	0.582
Body mass index (z-score)	−0.07 ± 1.14	−0.12 ± 1.24	−0.06 ± 1.10	0.554
Waist circumference (cm)	54.5 ± 7.0	53.9 ± 6.3	54.7 ± 7.2	0.214
Waist/height ratio	0.45 ± 0.04	0.45 ± 0.04	0.45 ± 0.04	0.532
Percentage body fat (%)	24.7 ± 7.9	23.9 ± 8.2	24.9 ± 7.7	0.170
Office systolic BP (mmHg)	103.9 ± 10.6	105.4 ± 11.5	103.4 ± 10.2	0.062
Office diastolic BP (mmHg)	64.4 ± 7.9	64.8 ± 7.1	64.3 ± 8.1	0.511
Office heart rate (bpm)	87.0 ± 12.9	89.3 ± 13.1	86.2 ± 11.5	0.009
Shuttle run (stages)	3.4 ± 1.6	3.1 ± 1.3	3.5 ± 1.7	0.041
Cortisol at baseline (mg/L)	0.18 ± 0.22	0.24 ± 0.20	0.15 ± 0.22	<0.001
Cortisol at pre (mg/L)	0.17 ± 0.19	0.28 ± 0.18	0.13 ± 0.18	<0.001
Cortisol at post (mg/L)	0.27 ± 0.27	0.13 ± 0.08	0.32 ± 0.30	<0.001

Values depicted as mean ± standard deviation (SD), or numbers and percentage. Baseline: Saliva sample in the morning (<08:00 a.m.); Pre: saliva sample before shuttle run; Post: saliva sample after shuttle run. Cortisol reactivity: change of cortisol concentration from pre to post shuttle run test. *p* value for comparison of percentile ranges.

**Table 2:** Saliva cortisol level at baseline, before and after the cardiorespiratory fitness test and cortisol reactivity in relation to clinical categories of body mass index, waist-to-height ratio, blood pressure and tertiles of physical fitness and ethnicity.

Parameter	*n*	Cortisol (ln [mg/L])			
		Baseline	Pre Sample	Post Sample	Change from Pre to Post
Body mass index					
normal weight	467	−1.99 (−2.05;−1.92) *	−2.08 (−2.1;−2.01)	−1.60 (−1.67;−1.53)	0.48 (0.49;0.57)
overweight	44	−2.04 (−2.23;−1.84)	−2.11 (−2.33;−1.89)	−1.52 (−1.75;−1.30)	0.59 (0.30;0.88)
obesity	40	−2.28 (−2.48;−2.07)	−2.18 (−2.41;−1.95)	−1.69 (−1.93;−1.45)	0.49 (0.19;0.80)
Body mass index ^a^					
normal weight	467	−2.00 (−2.06;−1.94)	−2.07 (−2.14;−2.00)	−1.61 (−1.67;−1.54)	0.47 (0.38;0.56)
overweight	44	−1.99 (−2.18;−1.80)	−2.14 (−2.36;−1.91)	−1.50 (−1.72;−1.28)	0.64 (0.35;0.93)
obesity	40	−2.13 (−2.33;−1.92)	−2.24 (−2.48;−2.00)	−1.67 (−1.91;−1.43)	0.57 (0.26;0.88)
Waist/height ratio					
<0.5	508	−1.98 (−2.03;−1.92) ***	−2.08 (2.15;−2.02)	−1.60 (−1.67;−1.54)	0.48 (0.40;0.57)
>0.5	43	−2.42 (−2.62;−2.22)	−2.18 (−2.41;−1.96)	−1.60 (−1.83;−1.37)	0.57 (0.28;0.86)
Waist/height ratio ^a^					
<0.5	508	−1.99 (−2.04;−1.94) *	−2.08 (−2.15;−2.02)	−1.60 (−1.67;−1.54)	0.48 (0.40;0.56)
>0.5	43	−2.25 (−2.45;−2.05)	−2.19 (−2.42;−1.95)	−1.59 (−1.82;−1.36)	0.60 (0.30;0.90)
Systolic BP					
normal BP	381	−2.01 (−2.07;−1.94)	−2.13 (−2.21;−2.06)	−1.58 (−1.66;−1.50)	0.56 (0.46;0.65)
elevated BP	60	−1.95 (−2.12;−1.78)	−2.01 (−2.20;−1.82)	−1.53 (−1.72;−1.34)	0.48 (0.24;0.73)
hypertension	110	−2.06 (−2.19;−1.94)	−1.99 (−2.13;−1.85)	−1.73 (−1.87;−1.58)	0.27 (0.08;0.45) *
Systolic BP ^b^					
normal BP	381	−2.03 (−2.09;−1.96)	−2.14 (−2.21;−2.07)	−1.56 (−1.64;−1.49)	0.58 (0.48;0.67)
elevated BP	60	−1.88 (−2.05;−1.72)	−2.01 (−2.20;−1.82)	−1.59 (−1.77;−1.40)	0.43 (0.18;0.67)
hypertension	110	−2.02 (−2.14;−1.90)	−1.96 (−2.11;−1.82)	−1.74 (−1.88;−1.60)	0.22 (0.04;0.40) **
Diastolic BP					
normal BP	429	−2.01 (−2.08;−1.95)	−2.10 (−2.17;−2.03)	−1.58 (−1.65;−1.51)	0.52 (0.43;0.61)
elevated BP	46	−2.09 (−2.28;−1.89)	−2.00 (−2.22;−1.79)	−1.82 (−2.04;−1.60)	0.18 (−0.10;0.46)
hypertension	76	−1.96 (−2.11;−1.81)	−2.10 (−2.27;−1.93)	−1.59 (−1.76; −1.41)	0.51 (0.30;0.73)
Diastolic BP ^b^					
normal BP	429	−2.01 (−2.07;−1.95)	−2.10 (−2.17;−2.03)	−1.59 (−1.65;−1.52)	0.52 (0.43;0.61)
elevated BP	46	−2.11 (−2.29;−1.93)	−2.00 (−2.21;−1.78)	−1.80 (−2.01;−1.59)	0.20 (−0.08;0.48)
hypertension	76	−1.97 (−2.11;−1.83)	−2.08 (−2.25;−1.92)	−1.58 (−1.75; −1.42)	0.50 (0.28;0.72)
Physical fitness					
low	209	−2.09 (−2.18;−2.00)	−2.13 (−2.23;−2.03)	−1.76 (−1.87;−1.67) ***	0.44 (0.30;0.57) ***
moderate	196	−1.92 (−2.01;−1.82)	−2.00 (−2.11;−1.90)	−1.62 (−1.73;−1.52)	0.36 (0.22;0.49)
high	146	−2.02 (−2.13;−1.92)	−2.15 (−2.27;−2.03)	−1.34 (−1.47;−1.21)	0.74 (0.58;0.90)
Physical fitness ^c^					
low	209	−2.09 (−2.18;−2.00)	−2.17 (−2.28;−2.07)	−1.76 (−1.87;−1.66) ***	0.41 (0.27;0.54) **
moderate	196	−1.94 (−2.03;−1.85)	−1.99 (−2.09;−1.89)	−1.61 (−1.72;−1.51)	0.38 (0.25;0.51)
high	146	−1.99 (−2.10;−1.88)	−2.11 (−2.23;−1.98)	−1.36 (−1.49;−1.23)	0.75 (0.59;0.92)
Ethnicity					
black	324	−1.83 (−1.90;−1.76) ***	−2.12 (−2.20;−2.03)	−1.74 (−1.82;−1.66) ***	0.38 (0.28;0.49) **
white	227	−2.27 (−2.35;−2.19)	−2.05 (−2.15;−1.95)	−1.41 (−1.50;−1.31)	0.64 (0.52;0.77)
Ethnicity ^d^					
black	324	−1.84 (−1.91;−1.77) ***	−2.13 (−2.22;−2.05)	−1.74 (−1.82;−1.66) ***	0.40 (0.29;0.50) **
white	227	−2.26 (−2.34;−2.17)	−2.03 (−2.13;−1.93)	−1.41 (−1.51;−1.31)	0.62 (0.49;0.75)

Body mass index categories incorporated age and sex. All other data adjusted for age and sex. BP; blood pressure; baseline: Saliva sample in the morning (<08:00 a.m.); pre: saliva sample before shuttle run; post: saliva sample after shuttle run. Values depicted as mean ± 95% confidence interval. *p* value for linear trend was calculated. Superscript symbol denotes significance for: * *p* < 0.05; ** *p* <0.01; *** *p* < 0.001. ^a^ Additionally adjusted for ethnicity, diastolic blood pressure and shuttle run. ^b^ Additionally adjusted for ethnicity, body mass index and shuttle run. ^c^ Additionally adjusted for ethnicity, body mass index, diastolic blood pressure. ^d^ Additionally adjusted for body mass index, diastolic blood pressure and shuttle run.

## Data Availability

The data presented in this study are available on request from the corresponding author.
